# Recycling of a selectable marker with a self-excisable plasmid in *Pichia pastoris*

**DOI:** 10.1038/s41598-017-11494-5

**Published:** 2017-09-11

**Authors:** Cheng Li, Ying Lin, Xueyun Zheng, Qingyan Yuan, Nuo Pang, Xihao Liao, Yuanyuan Huang, Xinying Zhang, Shuli Liang

**Affiliations:** 1 Guangdong Key Laboratory of Fermentation and Enzyme Engineering, School of Biology and Biological Engineering, South China University of Technology, Guangzhou, 510006 P. R. China; 2 Guangdong research center of Industrial enzyme and Green manufacturing technology, School of Biology and Biological Engineering, South China University of Technology, Guangzhou, 510006 P. R. China

## Abstract

*Pichia pastoris* is a widely used heterologous protein production workhorse. However, with its multiple genetic modifications to solve bottlenecks for heterologous protein productivity, *P. pastoris* lacks selectable markers. Existing selectable marker recycling plasmids have drawbacks (e.g., slow growth and conditional lethality). Here, zeocin-resistance marker recycling vectors were constructed using the Cre/*loxP* recombination system. The vectors were used to (i) knock in heterologous phytase, xylanase and lipase expression cassettes, (ii) increase the phytase, xylanase and lipase gene copy number to 13, 5, and 5, respectively, with vector introduction and (iii) engineer the secretion pathway by co-overexpressing secretion helper factors (Sly1p and Sec1p) without introducing selectable markers, giving a phytase field of 0.833 g/L. The vectors allow selectable marker recycling and would be a useful tool to engineer *P. pastoris* for high heterologous protein productivity.

## Introduction

The budding methylotrophic yeast *Pichia pastoris*, currently reclassified as *Komagataella phaffii*, has become a workhorse to produce large quantities of medically and industrially important proteins^1^. *P. pastoris* is a valuable production system because of its ability to grow to very high cell densities using minimal media, its ability to produce gram amounts of recombinant protein per liter of culture both intracellularly and in a secretory fashion, and the availability of the strong and tightly regulated promoter *AOX1* (P_*AOX1*_)^2^. Fermentation can be readily scaled to meet greater demand, and parameters influencing protein productivity and activity, such as the pH, aeration and carbon source feed rate, can be controlled^3^. Large quantities of heterologous proteins have been produced in *P. pastoris* for basic research as well as industrial applications^4, 5^. Moreover, *P. pastoris* has been ruled as a generally recognized as safe (GRAS) strain for use in food industries by the Food and Drug Administration (FDA)^6, 7^. To date, *P. pastoris* has been applied for the expression of many proteins used in the food and feed industry^8, 9^.

The copy number of expression cassettes introduced into *P. pastoris* remains one of the early bottlenecks affecting heterologous protein productivity. Increasingly, arranged expression genes of target proteins show a trend of promoting their host cells to produce higher levels of proteins^10, 11^. However, the achievable copy numbers are limited in the natural state: if the expression vector transformed into *P. pastoris* is a single copy, the probability of the emergence of multi-copy integration is approximately 10% and the copy number is not controllable^12^. To gain high copy numbers of heterologous genes, multi-copy integration, using several rounds of gradient antibiotic concentration screening of a large number of colonies, led to random copy numbers^13, 14^, but required significant time and effort.

Moreover, there are some other bottlenecks for heterologous protein productivity in *P. pastoris* ^15, 16^ (e.g., the poor secretion)^15, 17^. During protein secretion, at each trafficking step that requires soluble NSF (N-ethylmaleimide-sensitive factor) receptor (SNARE) complex formation involving the Sec1/Munc18 (SM) proteins, the cargo proteins are delivered by fusion of the membrane of transport vesicles and the target membrane^18^. Among the SM proteins, Sly1p regulates endoplasmic reticulum (ER)-Golgi trafficking and Sec1p interacts with the vesicle trafficking between the Golgi and cell membrane^19^. Engineering the secretion pathway may solve this bottleneck of poor secrection^20^. For pathway engineering, DNA transformation systems based on homologous recombination for integration into genomes are one of the most powerful genetic techniques and have been extensively used in research on *P. pastoris*. However, the multiple genetic modifications of *P. pastoris* have faced a shortage of selectable markers^20–26^. With limited selectable markers, it is hard to process further engineering to solve the bottlenecks in *P. pastoris*.

To solve this problem, some selectable marker recycling plasmids are currently in use^22, 27, 28^. The first one used the uracil biosynthetic genes *URA*3 or *URA*5 as counter-selectable markers^22^. Unfortunately, uracil auxotroph host strains grow slowly, even in the presence of uracil^20, 23^. The second, also based on counter-selection, used the *Escherichia coli* toxin gene *mazF* ^28^ or the maize mitochondrial gene T-*urf*13^27^. However, a nearly 250 bp unwanted repeat sequence was left in the genome after each marker was rescued for knock-in of a gene of interest using *mazF* ^28^, and the toxicity of the T-*urf*13 gene might cause conditional lethality for some gene deletions^22^.

Recently, a Cre/*loxP* recombination system has been widely employed in various organisms^29–31^. When two *loxP* sites are placed flanking a marker gene, Cre recombinase can excise the marker gene and leave a *loxP* sequence behind. The use of mutant *lox* sequences, such as *lox71* and *lox66* can help avoid potential recombination between the newly introduced *loxP* site and the former *loxP* site left in the genome^30^.

In this study, we describe new marker recycling vectors for *P. pastoris* based on the Cre/*loxP* system. We used the vectors (i) to recycle a zeocin-resistance marker, (ii) to increase target gene copy number by re-introducing vectors, and (iii) to engineer the secretion pathway by co-overexpressing the genes of SM proteins Sly1p and Sec1p to increase secretion of the heterologous protein phytase.

## Results

### Construction of self-excising vectors for *P. pastoris*

**Figure 1:**
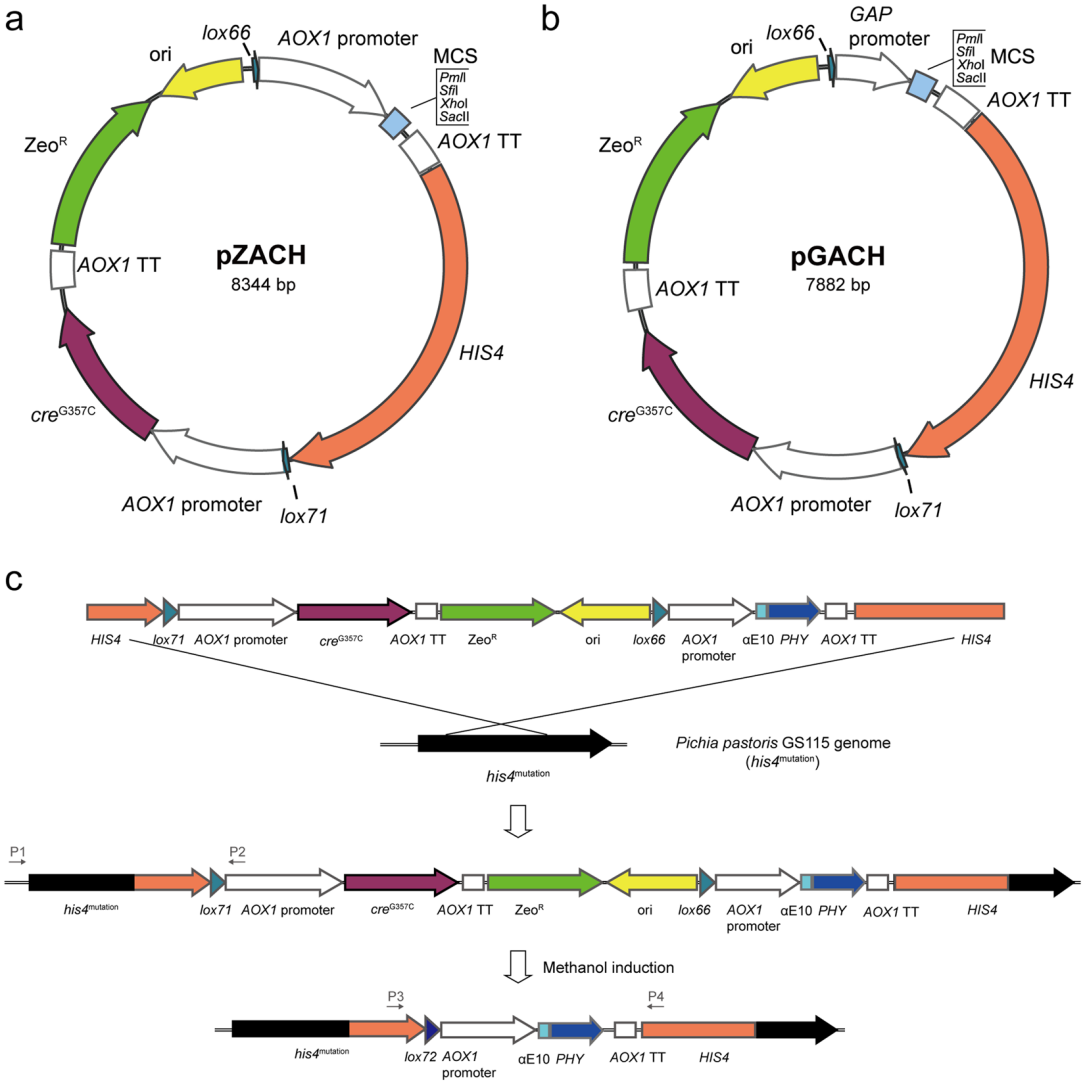
Schemes of Cre/*loxP* zeocin-resistance selectable marker recycling vectors. Filled arrows and boxes, plasmid elements: MCS, multiple unique cloning sites; *AOX1* TT, *AOX1* transcription termination; Zeo^R^, zeocin-resistance marker; ori, bacterial replication origin. (**a**) The vector using the *AOX1* promoter for gene expression; (**b**) vector using the *GAP* promoter for gene expression; (**c**) strategy for selectable marker recycling using the vectors (e.g., plasmid C-Phy). The linearized plasmid was introduced into *P. pastoris* cells by transformation and integrated into the genome through the *his4* locus. After methanol induction, the Cre-Zeo^R^ cassette was excised through recombination between *lox71* and *lox66*, leaving the double-mutant *lox72*. Chromosomal integrations and Cre-Zeo^R^ cassette excisions can be verified by PCR using primer pairs P1/P2 and P3/P4, respectively.

Using the Cre/*loxP* recombination system for zeocin-resistance (Zeo^R^) marker excision, two novel expression vectors, pZACH and pGACH (Fig. 1a,b), for *P. pastoris* genetic integration were constructed. These plasmids originated from the vectors pPICZA and pGAPZA (Invitrogen, Carlsbad, CA) containing *AOX1* and *GAP* promoter, respectively, which are two of most commonly used promoters in *P. pastoris* ^32^. First, the *cre* ^G357C^ gene, a silent mutation of *cre* that avoids the *Bam*HI site, was introduced into pPICZA. Second, the original Zeo^R^ cassette and ori region were replaced with the Cre-Zeo^R^ cassette with *lox71* and *lox66*, resulting in pZAC and pGAC. Third, the gene *HIS4*, used for homologous fragment insertion, was introduced into pZAC and pGAC, resulting in pZACH and pGACH.

The *cre* ^G357C^ was expressed with methanol induction under the control of the *AOX1* promoter, a tightly regulated promoter^2^. During zeocin resistance selection, the *AOX1* promoter was repressed on glucose-containing media. After shifting the cells to methanol-containing medium, *cre* ^G357C^ was expressed, leading to the recombination of the *lox71* and *lox66* sites, resulting in replacement of the Cre-Zeo^R^ cassette with *lox72* (Fig. 1c). Thus, the Zeo^R^ selection marker could be recycled in *P. pastoris*.

### Phytase, xylanase and lipase expression using the self-excising vectors

**Figure 2:**
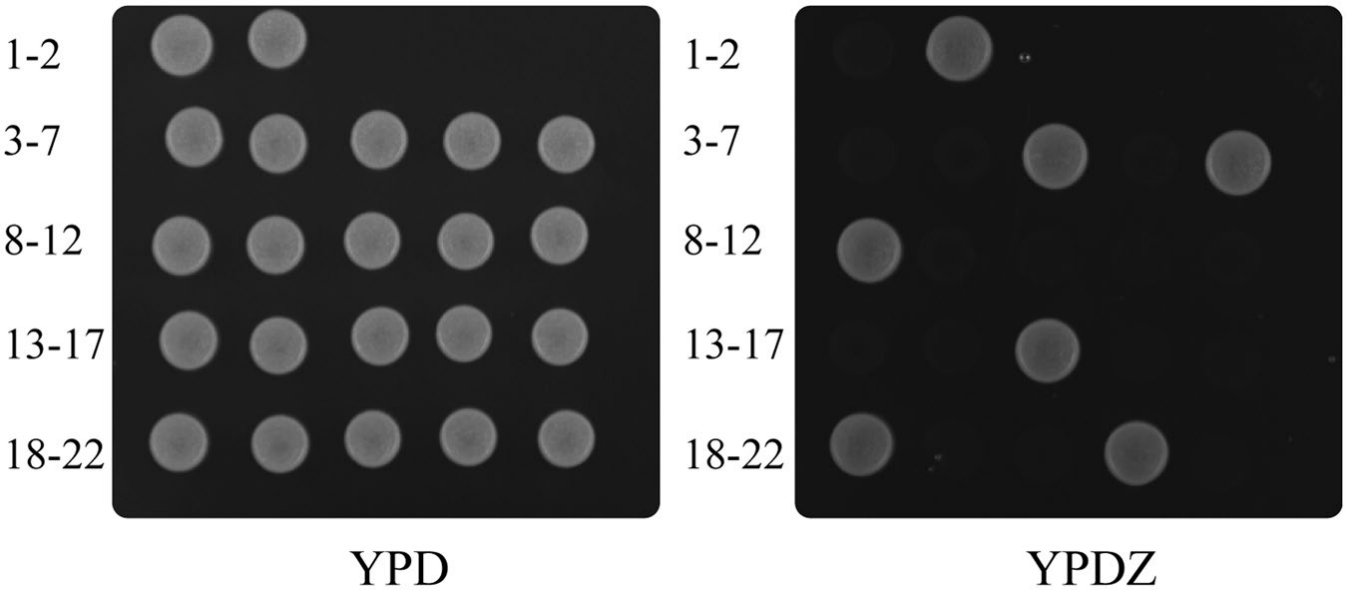
Assay of excision of the zeocin-resistance marker. Three different Zeo^R^ transformants were shifted to YPM for induction and then streaking on YPD plate. The isolated single colony from YPD plate was spotted on both YPD and YPDZ plates. (1) Wild type *P. pastoris* GS115; (2) the transformant without methanol induction; 3–22: 20 isolated single colonies from YPD plate after methanol induction. The experiments were repeated three times.

To test the Cre/*loxP* Zeo^R^ marker recycling vectors, the genes *PHY* that encodes the phytase from *Citrobacter amalonaticus* CGMCC 1696 (Phy)^33, 34^, *XYN* that encodes the xylanase A from *Bacillus halodurans* C-125 (Xyn)^10^ and *ARL* that encodes lipase from *Acinetobacter radioresistens* CMC-1 (Arl)^35^, which are three important kinds of enzymes applied in industry^10, 34–37^, were used as reporter genes. Considering the advantages of the most commonly used promoter P_*AOX1*_ for expression of heterologous proteins in *P. pastoris*, e.g., strong transcription level and tightly regulated (recently reviewed by Ahmad^38^), and advantages of the secreted expression system, e.g., simple purification, reduced potential degradation of heterologous proteins and toxicity to hosts by accumulation of secreted heterologous proteins^39, 40^, the vector pZACH was chosen for secreted heterologous proteins expression. Here, we used *PHY* as a detailed example for Zeo^R^ marker excision. The plasmid, C-Phy, was verified to be integrated into the genome by PCR with the primer pair P1 and P2 (Fig. 1c). Three different verified Zeo^R^ transformants were transferred to YPM liquid medium for induction. After the cultured YPM medium was streaked onto YPD plates (Supplementary Fig. S1), 20 colonies of each YPD plate were spotted on both YPD and YPDZ plates (Fig. 2). After methanol induction, nearly 30% of cells (experiments were repeated three times) retained the Zeo^R^ phenotype (Fig. 2). Nearly 100% of the colonies that lost the Zeo^R^ phenotype (Fig. 2) had excised the Zeo^R^ marker, as verified by PCR with the primer pair P3 and P4 (Fig. 1c and Supplementary Fig. S2, 3,384 bp). Sequencing of the PCR products confirmed that a *lox72* sequence remained after the Cre-Zeo^R^ cassette was excised. For the colonies that excised the Zeo^R^ marker, nearly 100% (experiments were reproduced three times) carried the *PHY* gene as detected by PCR using the primer pair Phy-S and Phy-A. It typically took 3 or 4 days to complete the Zeo^R^ marker excision (Supplementary Fig. S1). To determine the marker recycling frequencies, 10 Zeo^R^ transformants from each reporter gene were tested. Marker recycling frequencies were observed to be > 65% (Supplementary Table S2). This result is higher than that used T-*urf*13 as a counter-selectable marker (40%)^27^ and lower than that used *mazF* as a counter-selectable marker (>90%)^28^.

**Figure 3:**
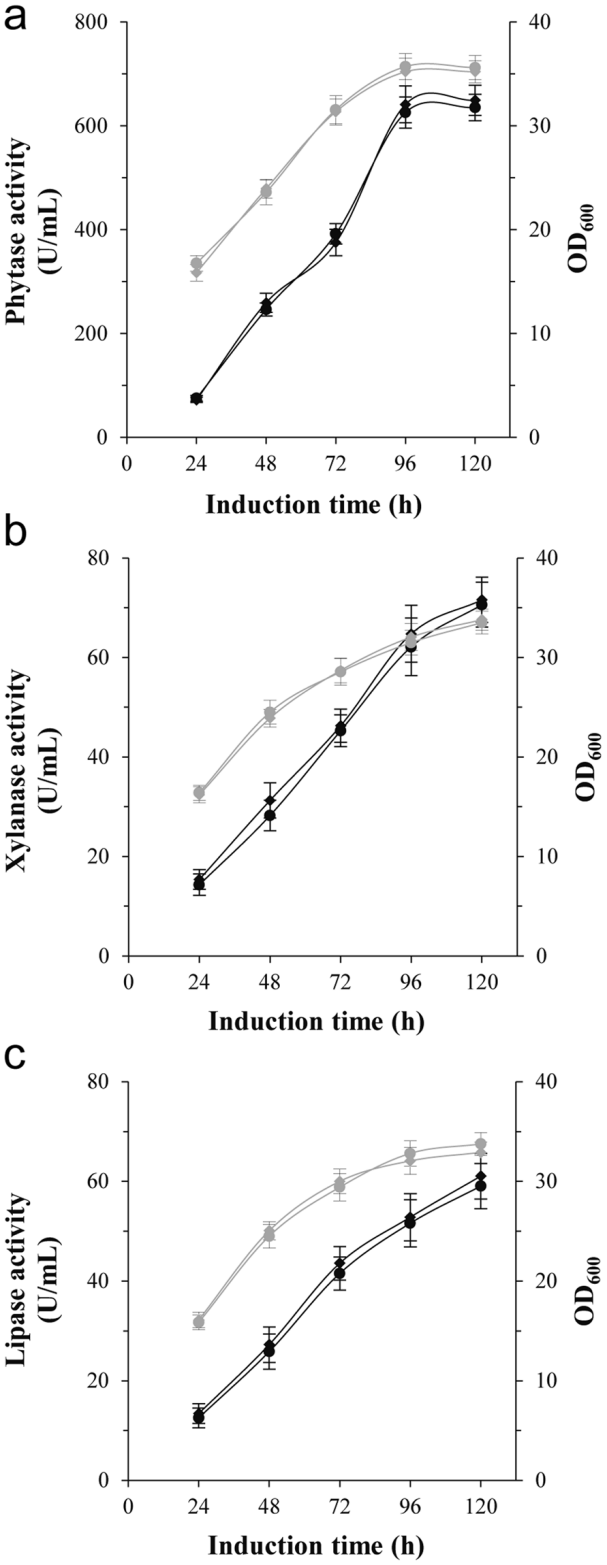
Expression of Phy, Xyn and Arl in *P. pastoris* using self-excising vectors. Time dependence of phytase (**a**), xylanase (**b**) or lipase (**c**) activity and cell growth after induction with methanol. Black lines indicate phytase (**a**), xylanase (**b**) or lipase (**c**) activity, and gray lines indicate OD_600_. (**a**) Rhombuses indicate GS115/C-Phy, and circles indicate the control GS115/αE10; (**b**) rhombuses indicate GS115/C-Xyn, and circles indicate the control GS115/pPICHKA-xynA; (**c**) rhombuses indicate GS115/C-Arl, and circles indicate the control GS115/pPICHKA-epARL.

The strains GS115/C-Phy, GS115/C-Xyn and GS115/C-Arl contained a single copy of the reporter gene, similar to the control strains (Table 1). During cultivation, these strains had similar growth and heterologous protein production as the control (Fig. 3a,b,c, Supplementary Table S3 and Supplementary Fig. S3). These results showed that the Cre/*loxP* Zeo^R^ marker recycling vectors could be used as the expression vector in *P. pastoris* hosts.

**Table 1:** Quantitative PCR assay of *PHY*, *XYN* and *ARL* copy numbers in the genomic DNA of recombinant yeast strains.

Strains	Reporter gene copy number in *P. pastoris* genome
GS115/αE10	0.996 ± 0.053
GS115/C-Phy	1.012 ± 0.024
GS115/pPICHKA-xynA	1.021 ± 0.023
GS115/C-Xyn	0.989 ± 0.025
GS115/pPICHKA-epARL	1.002 ± 0.031
GS115/C-Arl	1.031 ± 0.024
GS115/P-6c	5.986 ± 0.314
C-Phy/P-6c	7.089 ± 0.326
C-Phy/P-6c/P-6c	13.273 ± 0.212
GS115/X-4c	3.968 ± 0.241
C-Xyn/X-4c	5.015 ± 0.207
GS115/A-4c	4.016 ± 0.105
C-Arl/A-4c	4.986 ± 0.211

The threshold value (horizontal dashed line) was set at 0.2. Values indicate the average ± standard deviation from triplicate qPCR.

### Increased *PHY, XYN* and *ARL* gene copy number in *P. pastoris* using the self-excising vectors

**Figure 4:**
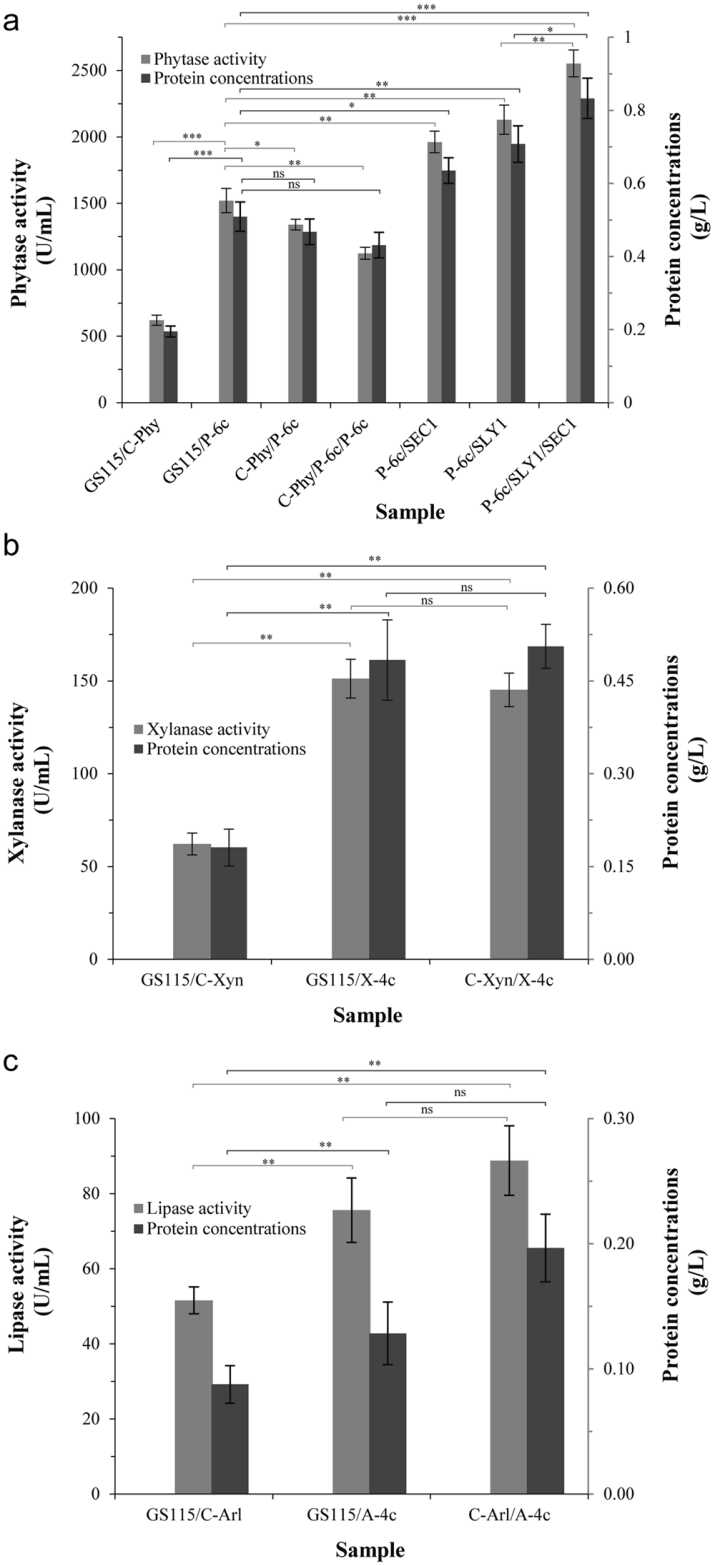
Increasing *PHY*, *XYN* and *ARL* copy numbers and co-overexpression of secretion helper factor in *P. pastoris* using self-excising vectors. (**a**) Effect of increasing *PHY* copy numbers and co-overexpression of *SLY1*, *SEC1*, or *SLY1* and *SEC1* on phytase production in recombinant strains carrying six *PHY* copies after a 96-h induction with methanol. Effect of increasing *XYN* (**b**) or *ARL* (**c**) copy numbers on Xyn (**b**) or Arl (**c**) activity and protein content after 96 h of induction with methanol. Statistical significance was examined using a two tailed by unpaired T-test analysis. *P < 0.05, **P < 0.01, ***P < 0.001, ns: no significant difference.

Gene copy number can influence recombinant protein productivity, and high gene copy numbers can enhance recombinant protein expression^11, 33, 35^. To test whether the Cre/*loxP* Zeo^R^ marker recycling vectors can be used to increase gene copy number directly, the vectors were re-introduced into the hosts. Here, we used *PHY* as a detailed example. Plasmid P-6c, containing six copies of *PHY*, was constructed. Based on strain GS115/C-Phy, P-6c was transformed using a Zeo^R^ selectable maker to form strain C-Phy/P-6c after Cre-Zeo^R^ cassette excision. Similarly, another P-6c was transformed into C-Phy/P-6c to form C-Phy/P-6c/P-6c. Similarly, strains GS115/X-4c, C-Xyn/X-4c, GS115/A-4c and C-Arl/A-4c were constructed. These strains were confirmed to contain desired copies of reporter genes (Table 1). These results showed that the Cre/*loxP* Zeo^R^ marker recycling vectors could be used to increase heterologous gene copy number by re-introducing the vectors. When the *ARL* copy number increased from 1 to 5, the lipase activity increased by 73% (Fig. 4c), while the xylanase activity increased by 134% from 1 to 5 (Fig. 4b). The phytase activity of strain GS115/P-6c increased by 145% (reaching 1521 U/mL, Fig. 4a) relative to the single-copy strain GS115/C-Phy, similar to a result described previously^33^. When *PHY* copy number increased to 7 and 13, the phytase activity decreased by 12% and 26% (Fig. 4a), which is in contrast to previously reported observations where excessive gene copy numbers can have a negative effect on recombinant protein productivity^15, 41^. Moreover, the transcription level of methanol utilization genes *AOX1*, *DAS1*, *DAK2* and *FBA1-2* decreased (Supplementary Fig. S4). This is in contrast to previously reported observations, where excessive copy numbers of a heterologous gene can down-regulate the transcription level of methanol utilization genes and lead to a slow methanol metabolism^42^. Moreover, folding and secretion of heterologous proteins have a high cost of ATP^43^ and strong heterologous protein expression would exhaust host cell metabolism, which could be overburdening to the host cell metabolism^44^, suggesting that excessive copy numbers of a heterologous gene may reflect a metabolic burden. Taken together, these results suggested that excessive gene copy numbers can be detrimental for heterologous protein productivity^11, 41^.

### Overexpression of the *SLY1* and *SEC1* genes using the self-excising vectors

The poor secretion of heterologous proteins might become a production bottleneck^17^. Increasing the expression of SM protein may solve this bottleneck^20^. Considering that pathway engineering strategies might further take advantage of fine-tuned constitutive promoters to ensure a controlled flux of metabolites^38^ and the potential negative effect that increased dosage of P_*AOX1*_ could lead to a slow methanol metabolism^42^, the vector pGAZH was used to co-overexpress *SLY1* and *SEC1* in the strain GS115/P-6c, respectively (Supplementary Fig. S5). Phytase activity increased by 40% and 29% (reaching 2,130 and 1,962 U/mL, Fig. 4a), whereas strain P-6c/GH (empty vector control) had similar phytase activity and protein content as strain GS115/P-6c. Consistent with co-overexpression of *SEC1* in strain P-6c/SLY1 (Supplementary Fig. S5), phytase activity and protein content increased by 20% and 18% (2,553 U/mL and 0.833 g/L, Fig. 4a and Supplementary Fig. S3), whereas strain P-6c/SLY1/GH (empty vector control) has similar phytase activity and protein content as strain P-6c/SLY1. With co-overexpression of *SLY1* and *SEC1*, phytase activity and protein content increased by 68% and 64% (Fig. 4a and Supplementary Fig. S3) compared with strain GS115/P-6c, similar to the effect of combined co-overexpression of *SLY1* and *SEC1* on α-amylase from *Aspergillus oryzae* in *S. cerevisiae* ^17^.

## Discussion

*P. pastoris* has widely been used as a cell factory to produce high titers of numerous recombinant proteins^4, 5^. Tools for genetic engineering in *P. pastoris* are widely available, but one limitation is the lack of selectable markers^20–26^. In this study, we constructed two vectors for zeocin-resistance marker recycling using the Cre/*loxP* recombination system. The vectors were used to express phytase from *C. amalonaticus* CGMCC 1696, xylanase A from *B. halodurans* C-125 and lipase from *A. radioresistens* CMC-1; to increase *PHY*, *XYN* and *ARL* copy numbers; and to co-overexpress the secretion helper factors, Sly1p and Sec1p, without introducing selectable markers.

The vectors have several advantages compared to other selectable marker recycling vectors in *P. pastoris*. First, only 34 bp (*lox72*) of unwanted sequence remained after each marker was rescued for knock-in of a gene of interest, compared to nearly 250 bp using the *mazF* counter-selection vector^28^. Second, the excised Zeo^R^ marker and remaining *lox72* did not influence the growth of *P. pastoris* when the strains grew slowly using *URA*3 or *URA*5 as counter-selectable markers^20, 23^. Third, only 3 or 4 days were required to complete selectable marker excision compared to 8 days or longer using *URA* markers^23^.

Using the vector in this study, only one transformation of *P. pastoris* cells was performed for antibiotic-resistance marker excision, and the recombinase gene *cre* ^G357C^ was excised when the antibiotic-resistance marker was excised. Compared with the method for recycling antibiotic-resistance marker using two plasmids^45^, our method saves time and effort for another transformation of *P. pastoris* cells and minimizes the risk of leaked expression of the remaining recombinase gene *cre*, which could lead to genetic instability of engineered *P. pastoris*.

In our previous study, a plasmid containing more than six tandem-repeats of *PHY* expression cassette (~18 kb) was difficult to construct *in vitro* ^33^. Using different selectable markers can also increase the target gene copy number^46^. However, the achieved copy number was limited, and to gain multiple integrated strains of target genes, we had to screen many antibiotic-marker-resistant colonies at gradient antibiotic concentrations^13, 14, 33^. These methods were not always successful^47^. Even after significant screening for higher copy gene integration strains at gradient antibiotic concentrations, excessive copy number can be detrimental for recombinant protein productivity in some cases^11, 33^. Using the selectable marker recycling vector in this study, we could increase target gene copy number *in vivo* by re-introducing the same expression vectors as many times as desired, and obtained higher copy gene integration strains than by constructing expression cassette tandem-repeats in plasmids^10^ or by using different selectable marker combinations^46^. We can evaluate whether there is a trend in which increasing recombinant protein gene copy number promoted protein productivity. When increasingly recombinant protein gene copy number becomes detrimental for recombinant protein productivity, re-introducing vectors can be stopped to save effort compared with screening large numbers of resistant colonies^14^.

By changing transgene copy number, protein secretion could become a bottleneck for heterologous protein expression^15, 17^. In some cases, secretory proteins are retained intracellularly with incomplete secretion^15^. Because of the limited selectable markers available, it is difficult to further engineer in *P. pastoris*. Here, enhancement of the secretion of Phy by co-overexpression of Sly1p and Sec1p was achieved using the zeocin-resistance marker recycling vector, suggesting that vesicle trafficking between the ER to Golgi and Golgi to cell membrane are the bottlenecks for Phy expression in *P. pastoris*. Furthermore, high recombinant protein expression may cause increasing accumulation of misfolded proteins, which causes ER stress and activates the unfolded protein response (UPR)^48, 49^. With the vector in this study, further engineering to improve the folding of recombinant proteins by overexpressing disulfide isomerase (Pdi1p)^50^ and/or endoplasmic reticulum oxidoreductin 1 (Ero1p)^51^ can be performed without introducing selectable markers. Enhancing the expression of one sole chaperone or transcription factor might shift the bottleneck to the next step^52^. Further pathway engineering can be performed to solve the bottlenecks of heterologous protein production using the vectors introduced in this study. These vectors provide a useful tool for pathway engineering to improve recombinant protein expression in *P. pastoris*.

Thus, the vectors in this study, using a Cre/*loxP* recombination system for zeocin-resistance selectable marker recycling, can be used to integrate a gene of interest and increase gene copy numbers. These will be useful tools for engineering *P. pastoris* for enhancing heterologous protein productivity.

## Methods

### Strains and growth conditions

*E. coli* TOP10 (Invitrogen, Carlsbad, CA) cells were grown at 37 °C in LB or low-salt LB (0.5% yeast extract, 1% trypton, and 1% or 0.5% NaCl) medium. Standard cloning procedures were performed in *E. coli* TOP10, as described by Sambrook & Fritsch^53^. Plasmid selection and maintenance was performed using 25 mg/L zeocin (Invitrogen) or 100 mg/L kanamycin (Invitrogen).

The *P. pastoris* strain GS115 (Invitrogen) was used to construct yeast strains. *P. pastoris* GS115 was cultured at 30 °C and 250 revolutions per minute (rpm) in YPD or YPM medium (1% yeast extract, 2% peptone, and 2% glucose or 1% methanol). BMGY or BMMY medium (1% yeast extract, 1.34% YNB, 2% peptone, 0.00004% biotin, 100 mM potassium phosphate (pH 6.0) and 1% glycerol or 1% methanol) was used for *P. pastoris* fermentation. Transformants were selected on YPDSZ plates (1% yeast extract, 2% glucose, 2% peptone, 18.2% sorbitol, 2% agar and 0.1 g/L zeocin).

Strains, vectors and primers used in this study are summarized in Supplementary Table S1.

### Construction of vectors

To avoid the *Bam*HI site in *cre*, the *cre* silent mutation *cre* ^G357C^ was amplified by PCR from the plasmid pSH47^54^ and cloned into pPICZA (Invitrogen) as follows: 5′ arms of *cre* ^G357C^ were amplified by PCR using the primer pair Cre-F and Cre-G357C-R and the 3′ arms using Cre-G357C-F and Cre-R. The *cre* ^G357C^ was generated using Cre-F and Cre-R, resulting in the vector pPICZA-cre^G357C^. To avoid the *Bam*HI site in the Cre-Zeo^R^ cassette of pPICZA-cre^G357C^, the 5′ arms of the Cre-Zeo^R^ cassette were amplified from pPICZA-cre^G357C^ using AOX1-lox71-F and AOXTT-A and the 3′ arms using AOXTT-F and Zeo_lox66_A. These two arms were assembled with fragments amplified from pPICZA or pGAPZA using AOX1-G-F and AOXTT-G-A or GAP-G-F and AOXTT-G-A using a Gibson Assembly Cloning kit (NEB, Boston, MA), resulting in the vectors pZAC and pGAC. To create the vectors pZACH and pGACH, *HIS4* was amplified from pPIC9k (Invitrogen) using HIS4-F and HIS4-R and was assembled with fragments amplified from pZAC or pGAC using 3AOX-F and 3AOX-R using the Gibson Assembly Cloning kit (NEB).

A phytase expression cassette from the plasmid pAOX1_d1+201_-αE10-phy-HKA (αE10)^33^ was ligated into the pZACH plasmid using *Bgl*II and *Bam*HI sites to create the vector pZACH-phy (C-Phy). Similarly, pZACH-xyn (C-Xyn) and pZACH-arl (C-Arl) were constructed based on pPICHKA-xynA^10^ and pPICHKA-epARL^35^. A similar method was used to obtain the six-copy plasmid pZACH-(phy)_6_ (P-6c), four-copy plasmid pZACH-(xyn)_4_ (X-4c) and pZACH-(arl)_4_ (A-4c) using the plasmid pPICZA-αE10-HKA/(Phy)_6_ (6c)^33^, pPICZA-(xynA)_4_^10^ and pPICHKA-(epARL)_4_^35^.

The genes *SLY1* [GenBank ID: CAY71482.1] and *SEC1* [GenBank ID: CAY71361.1] were obtained from *P. pastoris* strain GS115 genomic DNA using the appropriate primer pairs. All PCR products were ligated into the pGACH plasmid using *Pml*I-*Sac*II sites to create the vectors pGACH-SLY1 and pGACH-SEC1.

Restriction enzyme digestion and DNA sequencing assured that all plasmids matched their design.

### Yeast transformation and regeneration of selectable markers

Plasmids pZACH, C-Phy and P-6c were linearized with *Eam*1105I (Thermo Scientific, Waltham, MA) and transformed into *P. pastoris* GS115-competent cells using the electroporation method described by Cregg^47^. The transformed cells were selected on YPDSZ plates. The integration of these plasmids into the GS115 genome was verified by PCR using the appropriate primer pairs.

As shown in Supplementary Fig. S1, the Zeo^R^ transformants were shifted from YPDSZ plates to 5 mL YPM medium and grown for 20 h. Then, 20 μL of the YPM cultures was streaked onto YPD plates and incubated at 30 °C until colonies were visible (nearly 36 h). These colonies were picked, added to 10 μL sterilized water and spotted onto YPD and YPDZ (YPD plus 100 mg/L zeocin) plates. Colonies that grew on YPD plates but not on YPDZ plates indicated that the Cre-Zeo^R^ cassette might have been excised and that the selectable marker could be reused. The result was verified by PCR using appropriate primer pairs. After Cre-Zeo^R^ cassette excision, these strains formed GS115/pZACH, GS115/C-Phy and GS115/P-6c. Similarly, GS115/C-Xyn, GS115/C-Arl, GS115/X-4c, GS115/A-4c, C-Xyn/X-4c, C-Arl/A-4c, C-Phy/P-6c and C-Phy/P-6c/P-6c were constructed. A similar method was used to construct P-6c/GH, P-6c/SLY1, P-6c/SEC1, P-6c/SLY1/SEC1 and P-6c/SLY1/GH.

### *PHY*, *XYN* and *ARL* copy number determination and RT-PCR analysis

The quantitative PCR (qPCR) assay protocol was developed as described previously^33^. The standard plasmids pPICZαA-phy-G^33^, pPICZα-G-xynA^10^ and pPICHKA-AG^35^ consisted of a portion of the *GAPDH* gene sequence, which was used as the reference sequence because there is only a single copy in the *P. pastoris* genome^55^. Total RNA were extracted by the hot acidic phenol method^56^. Next, the cDNA synthesis was carried out using the PrimeScript™ Kit (TaKaRa, Shiga, Japan).

The qPCR and real-time PCR (RT-PCR) assays were repeated three times per sample. RT-PCR data were normalized using the *GAPDH* gene (i. e., housekeeping gene) as an endogenous control. The *PHY*, *XYN* or *ARL* copy number in each transformant was calculated using the Ct value of the genomic DNA and a standard curve.

### Shake flask cultivation of *P. pastoris* transformants

A single *P. pastoris* transformant was transferred into 5 mL of BMGY at 30 °C and 250 rpm for 20 h. The main cultures inoculated from precultures in BMGY to an initial optical density of 0.5 were collected by centrifugation and transferred into 20 mL of BMMY at 30 °C and 250 rpm. OD_600_ and phytase, xylanase or lipase activity were monitored and 1% (v/v) fresh methanol was added to BMMY every 24 h.

### Assay of phytase, xylanase and lipase activity

Phytase activity was determined as described previously^33^. The enzyme reaction mixture was preheated at 37 °C for 5 min. Next, 5.0 mM sodium phytate (pH 5.5) was added and the mixture was incubated at 37 °C for 30 min. The reaction was stopped by adding coloration solution. The absorbance of mixture was quantified at 415 nm.

Xylanase activity was determined as described previously^10^. An appropriately diluted enzyme source was added to 1% (w/v) beechwood xylan and incubated at 70 °C for 30 min. Then, the reaction was stopped by adding dinitrosalicylic acid reagent. The absorbance of the mixture was quantified at 540 nm.

Lipase activity was determined as described previously^35^. The enzyme reaction mixture was preheated at 55 °C for 5 min. Next, 0.05 M p-nitrophenyl caprylate was added and the mixture was incubated at 55 °C for 5 min. The produced p-nitrophenol in the reaction mixture was quantified at 405 nm.

All results were repeated thrice and used GS115/pZACH as background samples.

### SDS-PAGE, protein concentration and Western blot analysis

Samples (culture supernatants) were heated at 100 °C for 5 min in loading buffer. The mixture was subjected to 12% SDS-polyacrylamide gel electrophoresis (SDS-PAGE) in a Mini-gel system (Bio-Rad, Hercules, CA). The proteins in the gel were stained with Coomassie Brilliant Blue R-250 (Invitrogen).

The phytase protein concentration in the supernatants was analyzed by SDS-PAGE with bovine serum albumin (BSA; Invitrogen) as a standard. The phytase, xylanase or lipase and BSA bands in the gel were quantified by Quantity One (Bio-Rad). All results were reproduced three times.

Proteins in the SDS-PAGE gels were transferred to nitrocellulose (NC) membranes. The membranes were incubated with a mouse anti-FLAG (1:1000) or anti-HA monoclonal antibody (1:2000) and then exposed to the HRP-conjugated goat anti-mouse IgG monoclonal antibody. The protein bands were visualized by exposure using FluorChem M (ProteinSimple, San Jose, California).

### Statistical analysis

All data generated or analysed during this study are included in this published article (and its Supplementary Information files). Differences between groups were tested for statistical significance by using a two tailed by unpaired T-test in Microsoft Excel 2010 (Microsoft, Redmond, Washington). Differences were considered significant at *P* < 0.05.

## Electronic supplementary material

Supplementary information

## References

[1] Kurtzman CP (2009). Biotechnological strains of Komagataella (Pichia) pastoris are Komagataellaphaffii as determined from multigene sequence analysis. Journal of Industrial Microbiology & Biotechnology.

[2] Cregg, J. M., Barringer, K. J., Hessler, A. Y. & Madden, K. R. Pichia pastoris as a host system for transformations. *Molecular & Cellular Biology* **5**, 3376–3385 (1986). 10.1128/mcb.5.12.3376-3385.1985 PMC369166 3915774

[3] Higgins DR, Cregg JM (1998). Introduction to Pichia pastoris. Methods in Molecular Biology.

[4] Cregg JM, Cereghino JL, Shi J, Higgins DR (2000). Recombinant protein expression in Pichia pastoris. Molecular Biotechnology.

[5] Vogl T, Hartner FS, Glieder A (2013). New opportunities by synthetic biology for biopharmaceutical production in Pichia pastoris. Current Opinion in Biotechnology.

[6] Ciofalo, V., Barton, N. J., Coats, I. & Shanahan, D. Safety evaluation of a lipase enzyme preparation, expressed in Pichia pastoris, intended for use in the degumming of edible vegetable oil. **45**, 1–8 (2006). 10.1016/j.yrtph.2006.02.001 16563586

[7] Thompson CA (2010). FDA approves kallikrein inhibitor to treat hereditary angioedema. American journal of health-system pharmacy: AJHP: official journal of the American Society of Health-System Pharmacists.

[8] Spohner SC, Quitmann H, Czermak P (2015). Expression of enzymes for the usage in food and feed industry with Pichia pastoris. Journal of Biotechnology.

[9] Ravindran V, Son JH (2011). Feed enzyme technology: present status and future developments. Recent Patents on Food Nutrition & Agriculture.

[10] Lin XQ (2013). Bleach boosting effect of xylanase A from Bacillus halodurans C-125 in ECF bleaching of wheat straw pulp. Enzyme & Microbial Technology.

[11] Nordén K (2011). Increasing gene dosage greatly enhances recombinant expression of aquaporins in Pichia pastoris. Bmc Biotechnology.

[12] Lincereghino J, Cregg JM (2000). Heterologous protein expression in the methylotrophic yeast Pichia pastoris. FEMS Microbiology Reviews.

[13] Mellitzer A, Glieder A, Weis R, Reisinger C, Flicker K (2012). Sensitive high-throughput screening for the detection of reducing sugars. Biotechnology Journal.

[14] Marx H, Mecklenbräuker A, Gasser B, Sauer M, Mattanovich D (2009). Directed gene copy number amplification in Pichia pastoris by vector integration into the ribosomal DNA locus. FEMS Yeast Research.

[15] Idiris A, Tohda H, Kumagai H, Takegawa K (2010). Engineering of protein secretion in yeast: strategies and impact on protein production. Applied Microbiology and Biotechnology.

[16] Damasceno LM, Chung-Jr H, Batt CA (2012). Protein secretion in Pichia pastoris and advances in protein production. Applied Microbiology and Biotechnology.

[17] Hou J, Tyo K, Liu Z, Petranovic D, Nielsen J (2012). Engineering of vesicle trafficking improves heterologous protein secretion in Saccharomyces cerevisiae. Metabolic Engineering.

[18] Malsam J, Kreye S, Söllner TH (2008). Membrane fusion: SNAREs and regulation. Cellular & Molecular Life Sciences Cmls.

[19] Hashizume K, Cheng YS, Hutton JL, Chiu CH, Carr CM (2009). Yeast Sec1p functions before and after vesicle docking. Molecular Biology of the Cell.

[20] Lin CG (2001). New selectable marker/auxotrophic host strain combinations for molecular genetic manipulation of Pichia pastoris. Gene.

[21] Cereghino JL, Cregg JM (2000). Heterologous protein expression in the methylotrophic yeast Pichia pastoris. Fems Microbiology Reviews.

[22] Nett, J. H. & Gerngross, T. U. Cloning and disruption of thePpURA5 gene and construction of a set of integration vectors for the stable genetic modification ofPichia pastoris. **20**, 1279–1290 (2003). 10.1002/yea.1049 14618566

[23] Nett JH, Hodel N, Rausch S, Wildt S (2005). Cloning and disruption of the Pichia pastoris ARG1, ARG2, ARG3, HIS1, HIS2, HIS5, HIS6 genes and their use as auxotrophic markers. Yeast.

[24] Thor D (2005). Cloning and characterization of the Pichia pastoris MET2 gene as a selectable marker. Fems Yeast Research.

[25] Kimura M, Kamakura T, Tao QZ, Kaneko I, Yamaguchi I (1994). Cloning of the blasticidin S deaminase gene (BSD) from Aspergillus terreus and its use as a selectable marker for Schizosaccharomyces pombe and Pyricularia oryzae. Molecular Genetics and Genomics.

[26] Scorer CA, Clare JJ, Mccombie WR, Romanos MA, Sreekrishna K (1994). Rapid selection using G418 of high copy number transformants of Pichia pastoris for high-level foreign gene expression. Bio/technolgy.

[27] Soderholm, J., Bevis, B. J. & Glick, B. S. Vector for pop-in/pop-out gene replacement in Pichia pastoris. **31**, 306–310, 312 (2001). 10.2144/01312st01 11515366

[28] Yang J, Jiang W, Yang S (2009). mazF as a counter‐selectable marker for unmarked genetic modification of Pichia pastoris. Fems Yeast Research.

[29] Lambert JM, Bongers RS, Kleerebezem M (2007). Cre-lox-Based System for Multiple Gene Deletions and Selectable-Marker Removal in Lactobacillus plantarum. Applied & Environmental Microbiology.

[30] Pan R (2011). Sequential deletion of Pichia pastoris genes by a self-excisable cassette. Fems Yeast Research.

[31] Gueldener U, Heinisch J, Koehler GJ, Voss D, Hegemann JH (2002). A second set of loxP marker cassettes for Cre-mediated multiple gene knockouts in budding yeast. Nucleic Acids Research.

[32] Lincereghino J, Lincereghino GP (2007). Vectors and Strains for Expression. Methods Mol Biol.

[33] Li C (2015). Combined strategies for improving expression of Citrobacter amalonaticus phytase in Pichia pastoris. Bmc Biotechnology.

[34] Cheng L, Ying L, Huang Y, Liu X, Liang S (2014). Citrobacter amalonaticus Phytase on the Cell Surface of Pichia pastoris Exhibits High pH Stability as a Promising Potential Feed Supplement. Plos One.

[35] Zhao X (2013). Combined strategies for improving the heterologous expression of an alkaline lipase from Acinetobacter radioresistens CMC-1 in Pichia pastoris. Process Biochemistry.

[36] Pahoja VM, Sethar MA (2002). A Review of Enzymatic Properties of Lipase in Plants, Animals and Microorganisms. Journal of Applied Sciences.

[37] Honda H, Kudo T, Ikura Y, Horikoshi K (2011). Two types of xylanases of alkalophilic Bacillus sp. No. C-125. Canadian Journal of Microbiology.

[38] Ahmad M, Hirz M, Pichler H, Schwab H (2014). Protein expression in Pichia pastoris: recent achievements and perspectives for heterologous protein production. Applied Microbiology and Biotechnology.

[39] Cregg JM, Vedvick TS, Raschke WC (1993). Recent Advances in the Expression of Foreign Genes in Pichia pastoris. Bio/technology.

[40] Makrides SC (1996). Strategies for achieving high-level expression of genes in Escherichia coli. Microbiological reviews.

[41] Hohenblum H, Gasser B, Maurer M, Borth N, Mattanovich D (2004). Effects of gene dosage, promoters, and substrates on unfolded protein stress of recombinant Pichia pastoris. Biotechnology and bioengineering.

[42] Cámara E (2017). Increased dosage of AOX1 promoter-regulated expression cassettes leads to transcription attenuation of the methanol metabolism in Pichia pastoris. Scientific Reports.

[43] Tyo KE, Liu Z, Petranovic D, Nielsen J (2012). Imbalance of heterologous protein folding and disulfide bond formation rates yields runaway oxidative stress. BMC Biology.

[44] Kramer W, Elmecker G, Weik R, Mattanovich D, Bayer K (1996). Kinetic Studies for the Optimization of Recombinant Protein Formation. Annals of the New York Academy of Sciences.

[45] Gasser B, Dragosits M, Mattanovich D (2010). Engineering of biotin-prototrophy in Pichia pastoris for robust production processes. Metabolic Engineering.

[46] Williams KE, Jiang J, Ju J, Olsen DR (2008). Novel strategies for increased copy number and expression of recombinant human gelatin in Pichia pastoris with two antibiotic markers. Enzyme & Microbial Technology.

[47] Cregg JM (2009). Expression in the yeast Pichia pastoris. Methods in Enzymology.

[48] Whyteside G (2011). Native-state stability determines the extent of degradation relative to secretion of protein variants from Pichia pastoris. Plos One.

[49] Cudna RE, Dickson AJ (2003). Endoplasmic reticulum signaling as a determinant of recombinant protein expression. Biotechnology & Bioengineering.

[50] Shusta EV, Raines RT, Plückthun A, Wittrup KD (1998). Increasing the secretory capacity of Saccharomyces cerevisiae for production of single-chain antibody fragments. Nature Biotechnology.

[51] Lloyd, R. Method for Producing Natively Folded Proteins in a Prokaryotic Host. *Oulun Yliopisto* (2016).

[52] Delic M, Göngrich R, Mattanovich D, Gasser B (2014). Engineering of protein folding and secretion-strategies to overcome bottlenecks for efficient production of recombinant proteins. Antioxidants & Redox Signaling.

[53] Sambrook, J., Fritsch, E. F. & Maniatis, T. *Molecular cloning: a laboratory manual*. (CSH, 1989).

[54] Güldener U, Heck S, Fielder T, Beinhauer J, Hegemann JH (1996). A new efficient gene disruption cassette for repeated use in budding yeast. Nucleic Acids Research.

[55] Waterham HR, Digan ME, Koutz PJ, Lair SV, Cregg JM (1997). Isolation of the Pichia pastoris glyceraldehyde-3-phosphate dehydrogenase gene and regulation and use of its promoter. Gene.

[56] Köhrer K, Domdey H (1991). Preparation of high molecular weight RNA. Methods in Enzymology.

