# Supplementary material for: Recycling of a selectable marker with a self-excisable plasmid in Pichia pastoris
Source: Sci Rep. 2017 Sep 11;7:11113. doi: 10.1038/s41598-017-11494-5 (PMC5593967; doi:10.1038/s41598-017-11494-5)
Supplement: Supplementary file 1 — Supplementary information [file 41598_2017_11494_MOESM1_ESM.pdf]

Supplementary Information

Recycling of a selectable marker with a self-excisable plasmid in *Pichia pastoris*

Cheng Li[1, 2], Ying Lin[1, 2], Xueyun Zheng[1, 2], Qingyan Yuan[1, 2], Nuo Pang[1, 2] , Xihao Liao[1, 2], Yuanyuan Huang[1, 2], Xinying Zhang[1, 2], Shuli Liang[1, 2]*

1. Guangdong Key Laboratory of Fermentation and Enzyme Engineering, School of Biology and Biological Engineering, South China University of Technology, Guangzhou 510006, P. R. China

2. Guangdong research center of Industrial enzyme and Green manufacturing technology, School of Biology and Biological Engineering, South China University of Technology, Guangzhou, 510006, P. R. China

*To whom correspondence should be addressed. Shuli Liang, Tel: +86 20 39380605; Fax: +86 20 39380605; E-mail: shuli@scut.edu.cn

Tables and Figures

Table S1 3-6

Table S2 7

Table S3 8

Figure S1 …..9

Figure S2 10

Figure S3 11

Figure S4 12

Figure S5 13

Table S1. Primers, vectors and strains used in this study.
The restriction sites in the primer sequences are underlined.

	**Primer sequences (5’-3’) or a short description of the plasmids**	**Endonucleases**
Cre-F	GCTGAATTCATGTCCAATTTACTGACCG	*Eco*RI
Cre-G357C-R	CTTTTCGGATGCGCCGCATAACCAG	
Cre-G357C-F	CTGGTTATGCGGCGCATCCGAAAAG	
Cre-R	CATGGGCCCCTAATCGCCATCTTCCAGC	*Apa*I
AOX1-lox71-F	CAATACCGTTCGTATAGCATACATTATACGAAGTTATTAACATCCAAAGACGAAAGGTTGAATG	
AOXTT-A	TGGGGGTTCCGCACAAACGAAGGTC	
AOXTT-F	TTTGTGCGGAACCCCCACACACCATAG	
Zeo_lox66_A	GATCTTACCGTTCGTATAATGTATGCTATACGAAGTTATGATCTCATGACCAAAATCCC	
AOX1-G-F	ATACGAACGGTAAGATCTAACATCCAAAGACG	
GAP-G-F	ATACGAACGGTAAGATCTTTTTTGTAGAAATG	
AOXTT-G-A	CTATACGAACGGTATTGAAGCTATGGTGTGTGGG	
HIS4-F	ATGCTATACGAACGGTATTAAATAAGTCCCAGTTTCT	
HIS4-R	TTCGTTTGTGCGGATCCATGACATTTCCCTTGCTACC	
3AOX-F	TACCGTTCGTATAGCATACA	
3AOX-R	GGATCCGCACAAACGAAGGT	
SLY1-F	CGTGGTACCATGCTTCATTTGAATG	*Pml*I
SLY1-R	GATCCGCGGTTACTTATCGTCATCATCCTTGTAATCTTTTGCTTCGGCA	*Sac*II
SEC1-F	CGTGGTACCATGTACCCATACGATGTTCCAGATTACGCTGCTTCTGATCTGATTAA	*Pml*I
SEC1-R	GATCCGCGGCTATTTCCAAAATTTCTTCA	*Sac*II
RT-G1	GTCGGGACACGCCTGAAACT	

RT-G2		CCACCTTTTGGACCCTATTGAC
RT-Phy1		TGGTTGGGGTAGAATCAC
RT-Phy2		TGCTTCTGAGGAGGATGA
Prtx1		AGGTGCTGATAAGATTGT
Prtx2		TTGCTTGTTAGCCTTT
PrtA1		GAGAAATACCGCTCCTAC
PrtA2		GCAACATACTTCCACCAA
P1		TCAGATCTCCTGATGACTGA
P2		TTTCTTTTTGACCAACTGGC
P3		GTACCAACCACACTTTGCCA
P4		CGATCAGCTCCTCAAATTGG
Phy-S		GAGGTTCCAGACGATATGAAATTG
Phy-A		TCTATTAACGTCGGCCA
RT-AOX1-1		GAGAGTTCTTCTGGTGTTGG
RT-AOX1-2		GAATCAAGCCTCAGTACGAG
RT-DAK2-1		GGCTGGAACTTCTTTAGTGC
RT-DAK2-2		TGTTTGCTCTGGCTGGAATG
RT-DAS1-1		TTGGTTCGGTAGGCTTGTCT
RT-DAS1-2		CCAGCTAAAGGTGACGAGTT
RT-FBA1-2-1		GCACCTTCTTATGGCATACC
RT-FBA1-2-2		TTCATCGTCTGTTTCCTCCG
**Plasmids**	**Plasmids (abbreviation)**	
pPICZA		Invitrogen
pGAPZA		Invitrogen
pPIC9K		To offer the *HIS4* gene fragment
pAOX1$_{d1+201}$-αE10-phy-HKA	αE10	To offer the phytase expression cassette

pPICHKA-xynA		To offer the xylanase expression cassette
pPICHKA-epARL		To offer the lipase expression cassette
pPICZA-$(xynA)_4$		To offer four copies of xylanase expression cassettes
pPICZA-αE10-HKA/$(Phy)_6$	6c	To offer six copies of phytase expression cassettes
pPICHKA-$(epARL)_4$		To offer four copies of lipase expression cassettes
pPICZαA-phy-G		To detect *PHY* gene copy number
pPICZα-G-xynA		To detect *XYN* gene copy number
pPICHKA-AG		To detect *ARL* gene copy number
pPICZA-cre^{G357C}		To express the Cre recombinase
pZAC		Cre/*loxP* zeocin-resistance recycling vectors with *AOX1* promoter
pGAC		Cre/*loxP* zeocin-resistance recycling vectors with *GAP* promoter
pZACH		Cre/*loxP* zeocin-resistance recycling vectors with *AOX1* promoter and *HIS4*
pGACH		Cre/*loxP* zeocin-resistance recycling vectors with *GAP* promoter and *HIS4*
pZACH-phy	C-Phy	To express *PHY* using Cre/*loxP* zeocin-resistance marker recycling vectors
pZACH-$(phy)_6$	P-6c	To increase *PHY* gene copy number using Cre/*loxP* zeocin-resistance marker recycling vectors
pZACH-xyn	C-Xyn	To express *XYN* using Cre/*loxP* zeocin-resistance marker recycling vectors
pZACH-$(xyn)_4$	X-4c	To increase *XYN* gene copy number using Cre/*loxP* zeocin-resistance marker recycling vectors
pZACH-arl	C-Arl	To express *ARL* using Cre/*loxP* zeocin-resistance marker recycling vectors
pZACH-$(arl)_4$	A-4c	To increase *ARL* gene copy number using Cre/*loxP* zeocin-resistance marker recycling vectors
pGACH-SLY1		To overexpress Sly1p using Cre/*loxP* zeocin-resistance marker recycling vectors
pGACH-SEC1		To overexpress Sec1p using Cre/*loxP* zeocin-resistance marker recycling vectors
**Strains**	**Strains (abbreviation)**	
Escherichia coli TOP10		Invitrogen
Pichia pastoris GS115		Invitrogen
GS115/$pAOX1_{d1+201}$-αE10-phy-HKA	GS115/αE10	For secreted expression of Phy
GS115/pPICHKA-xynA		For secreted expression of Xyn

GS115/pZACH-arl		For secreted expression of Arl
GS115/pZACH		Control
GS115/pZACH-phy	GS115/C-Phy	For secreted expression of Phy after zeocin-resistance marker excised
GS115/pZACH-xyn	GS115/C-Xyn	For secreted expression of Xyn after zeocin-resistance marker excised
GS115/pZACH-arl	GS115/C-Arl	For secreted expression of Arl after zeocin-resistance marker excised
GS115/pZACH-$(xyn)_4$	GS115/X-4c	For secreted expression of Xyn using 4 *XYN* expression cassettes after zeocin-resistance marker excision
GS115/pZACH-$(arl)_4$	GS115/A-4c	For secreted expression of Arl using 4 *ARL* expression cassettes after zeocin-resistance marker excision
pZACH-xyn/pZACH-$(xyn)_4$	C-Xyn/X-4c	For secreted expression of Xyn using 5 *XYN* expression cassettes after zeocin-resistance marker excision
pZACH-arl/pZACH-$(arl)_4$	C-Arl/A-4c	For secreted expression of Arl using 5 *ARL* expression cassettes after zeocin-resistance marker excision
GS115/pZACH-$(phy)_6$	GS115/P-6c	For secreted expression of Phy using 6 *PHY* expression cassettes after zeocin-resistance marker excision
pZACH-phy/pZACH-$(phy)_6$	C-Phy/P-6c	For secreted expression of Phy using 7 *PHY* expression cassettes after zeocin-resistance marker excision
pZACH-phy/pZACH-$(phy)_6$/pZACH-$(phy)_6$	C-Phy/P-6c/P-6c	For secreted expression of Phy using 13 *PHY* expression cassettes after zeocin-resistance marker excision
pZACH-$(phy)_6$/pGACH	P-6c/GH	Control
pZACH-$(phy)_6$/pGACH-SLY1	P-6c/SLY1	For secreted expression of phytase using 6 *PHY* expression cassettes and overexpression of Sly1p after zeocin-resistance marker excision
pZACH-$(phy)_6$/pGACH-SEC1	P-6c/SEC1	For secreted expression of phytase using 6 *PHY* expression cassettes and overexpression of Sec1p after zeocin-resistance marker excision
pZACH-$(phy)_6$/pGACH-SLY1/pGACH	P-6c/SLY1/GH	Control
pZACH-$(phy)_6$/pGACH-SLY1/pGACH-SEC1	P-6c/SLY1/SEC1	For secreted expression of phytase using 6 *PHY* expression cassettes and overexpression of Sly1p and Sec1p after zeocin-resistance marker excision

Table S2. Marker recycling frequencies.
For each transformations of different plasmids, 10 Zeo^R transformants were test. Marker-recycling frequencies: the rate of colonies excised Zeo^R marker verified by plates spotting multiplied by the rate of colonies excised Zeo^R marker verified by PCR.

Plasmids	Marker recycling frequencies (%)
C-Phy	72 ±3.4
C-Arl	68 ±2.1
C-Xyn	70 ±3.4

Table S3. Protein concentrations of strains contained one copy of different reporter genes after 96 h of induction with methanol.

Strains	Protein concentrations (mg/mL)
GS115/αE10	0.190 ±0.021
GS115/C-Phy	0.195 ±0.027
GS115/pPICHKA-xynA	0.196 ±0.020
GS115/C-Xyn	0.191 ±0.015
GS115/pPICHKA-epARL	0.089 ±0.018
GS115/C-Arl	0.093 ±0.014

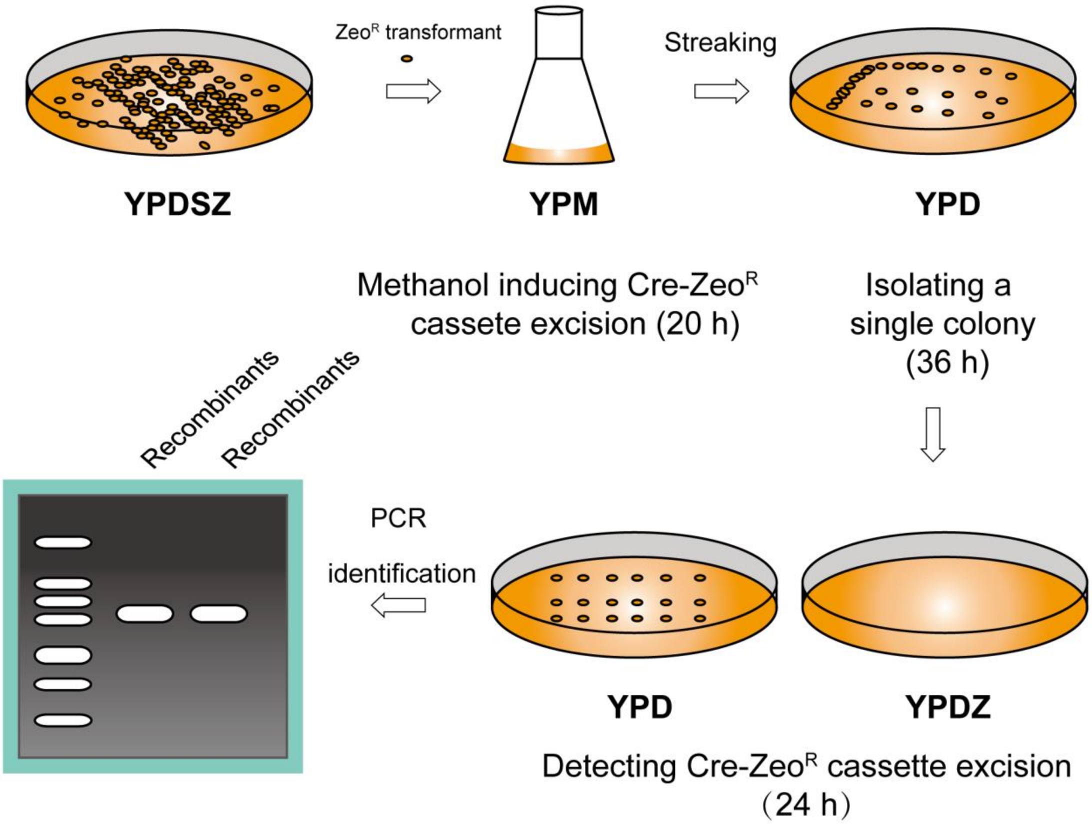

Figure S1. Schematic of strategy for zeocin-resistance marker excision.

The Zeo^R transformants were shifted from YPDSZ plates to YPM. After methanol induction, YPM cultures were streaked onto YPD plates. The single colony from last step was spotted on both YPD and YPDZ plates. Colonies that can grow on the YPD plate but not YPDZ plate, which indicated that the Cre-Zeo^R cassette might have been excised. The result will be verified by PCR using primers pairs P3 and P4.

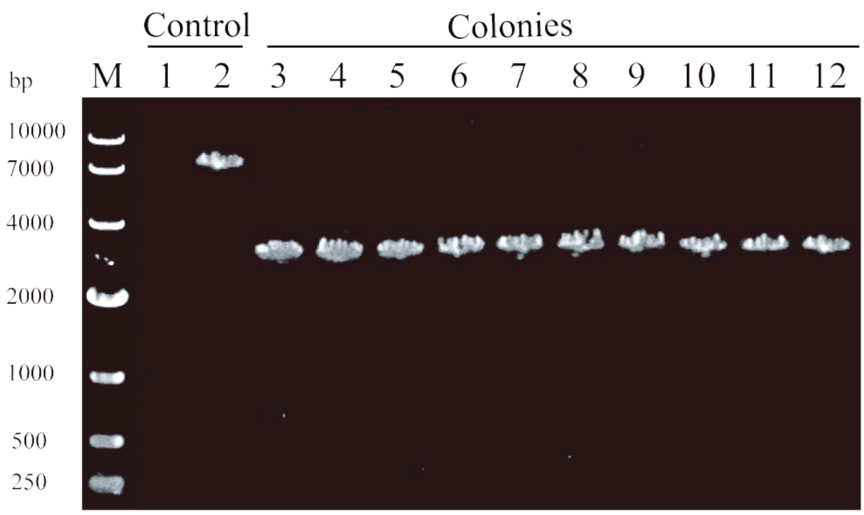

Figure S2. PCR Assay of excision of the zeocin-resistance marker using primer pair P3 and P4.

Lane M: DNA marker; lane 1: control (wild type *P. pastoris* GS115); lane 2: the transformant without methanol induction; lanes 3-12: colonies can grow on YPD but not YPDZ in Figure 2.

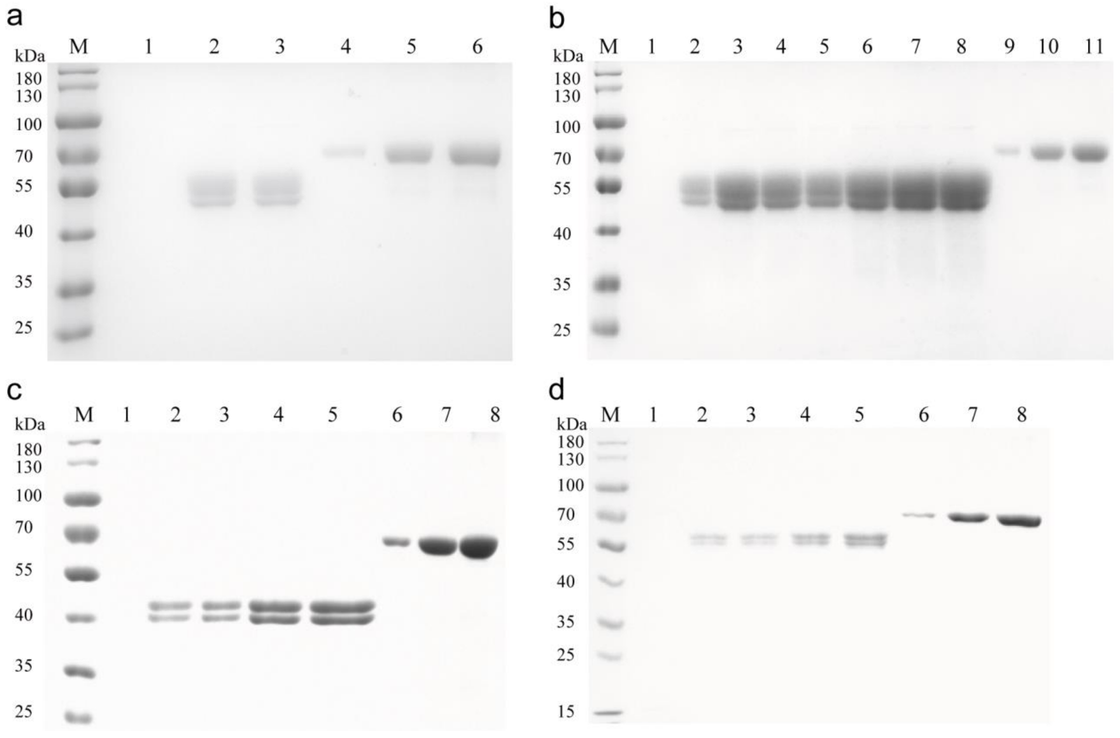

Figure S3. SDS-PAGE analysis of Phy, Xyn and Arl in *P. pastoris*

SDS-PAGE analysis of the culture supernatant containing Phy (a and b), Xyn (c) and Arl (d) (stained with Coomassie Blue) after methanol induction for 96 h. (a) Lane M: Protein marker; lane 1: GS115/pZACH; lane 2: GS115/αE10; lane 3: GS115/C-Phy; lane 4: 0.05 mg/mL BSA; lane 5: 0.2 mg/mL BSA; lane 6: 0.3 mg/mL BSA. (b) Lane M: protein marker; lane 1: GS115/pZACH; lane 2: GS115/C-Phy; lane 3: GS115/P-6c; lane 4: C-Phy/P-6c; lane 5: C-Phy/P-6c/P-6c; lane 6: P-6c/SEC1; lane 7: P-6c/SLY1; lane 8: P-6c/SLY1/SEC1; lane 9: 0.05 mg/mL BSA; lane 10: 0.2 mg/mL BSA; lane 11: 0.3 mg/mL BSA. (c) Lane M: Protein marker; lane 1: GS115/pZACH; lane 2: GS115/pPICHKA-xynA; lane 3: GS115/C-Xyn; lane 4: GS115/X-4c; lane 5: C-Xyn/X-4c; lane 6: 0.05 mg/mL BSA; lane 7: 0.2 mg/mL BSA; lane 8: 0.3 mg/mL BSA. (d) Lane M: Protein marker; lane 1: GS115/pZACH; lane 2: GS115/pPICHKA-epARL; lane 3: GS115/C-Arl; lane 4: GS115/A-4c; lane 5: C-Arl/A-4c; lane 6: 0.05 mg/mL BSA; lane 7: 0.2 mg/mL BSA; lane 8: 0.3 mg/mL BSA.

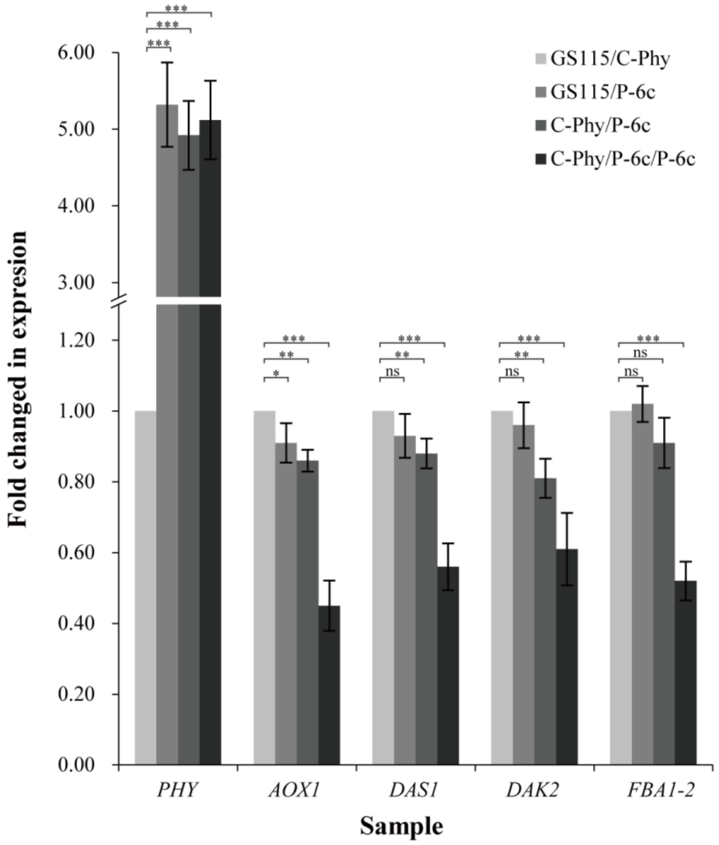

Figure S4. Excessive copy numbers of *PHY* down-regulated the transcription level of methanol utilization genes.

Transcription levels of *PHY* and four methanol utilization related genes in the different *P. pastoris* strains. All four strains were harvested after 96 h of methanol induction. Subsequently, mRNA levels were analyzed by real-time PCR. *PHY* and four methanol utilization related genes (*AOX1*, *DAS1*, *DAK2* and *FBA1-2*) levels were normalized relative to GS115/C-Phy. Statistical significance was examined using a two tailed by unpaired T-test analysis. *$P < 0.05$, **$P < 0.01$, ***$P < 0.001$, ns: no significant difference.

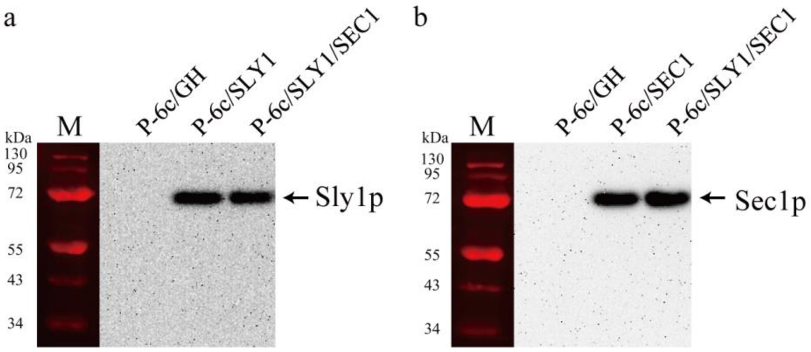

Figure S5. Western blot analysis of Sly1p and Sec1p.

All four strains were harvested after 96 h of methanol induction. Subsequently, the Western blot analysis of Sly1p (a) and Sec1p (b) used anti-FLAG and anti-HA antibody, respectively.
